# Mitogen-activated protein kinases, Fus3 and Kss1, regulate chronological lifespan in yeast

**DOI:** 10.18632/aging.101350

**Published:** 2017-12-21

**Authors:** Maneesha Aluru, Tori McKinney, Anne-Kathryn L. Venero, Shilpa Choudhury, Matthew Torres

**Affiliations:** ^1^ Georgia Institute of Technology, School of Biological Sciences, Atlanta, GA 30332, USA

**Keywords:** MAPK, CLS, Tor1, Fus3, gene network

## Abstract

Using a systems-based approach, we have identified several genes not previously evaluated for a role(s) in chronological aging. Here, we have thoroughly investigated the chronological lifespan (CLS) of three of these genes (*FUS3*, *KSS1* and *HOG1*) and their protein products, each of which have well-defined cell signaling roles in young cells. The importance of *FUS3* and *KSS1* in CLS are largely unknown and analyzed here for the first time. Using both qualitative and quantitative CLS assays, we show that deletion of any of the three MAPK's increases yeast lifespan. Furthermore, combined deletion of any *MAPK* and *TOR1, most* prominently *fus3Δ/tor1Δ,* produces a two-stage CLS response ending in lifespan increase greater than that of *tor1Δ*. Similar effects are achieved upon endogenous expression of a non-activatable form of Fus3. We speculate that the autophagy-promoting role of *FUS3*, which is inherently antagonistic to the role of *TOR1*, may in part be responsible for the differential aging phenotype of *fus3Δ/tor1Δ*. Consistent with this notion we show that nitrogen starvation, which promotes autophagy by deactivating Tor1, results in decreased CLS if *FUS3* is deleted. Taken together, these results reveal a previously unrealized effect of mating-specific MAPKs in the chronological lifespan of yeast.

## INTRODUCTION

Aging, or the gradual decline in function of cellular processes that naturally occur over the lifespan of an organism, is a complex biological phenomenon impacted by genetic and environmental factors and is a major risk factor for human disease. Several hallmarks of aging have been well documented, most of which involve the ability of cells to prevent or repair cellular damage and/or the ability to process or store nutrients [1]. In addition, altered intracellular communication mediated by changes in the organization of protein pathways and networks also define the aging profile of cells [2]. Thus, understanding the biology of aged cells cannot rely completely on models derived from young cells, but requires rediscovery of how proteins, path-ways and networks change throughout the aging process.

The budding yeast *Saccharomyces cerevisiae* has long served as one of the most powerful model organisms for identifying genes fundamental to the process of aging, many of which have later been found to also be important in mammals [3]. Indeed, the genetic tractability of budding yeast coupled with the variety of techniques and tools – both *in vivo* and *in silico* – have led to the discovery of several principal mechanisms of longevity that are, in some cases, conserved in mammals. Furthermore, large-scale screening methods enabling quantitative analysis of longevity has resulted in the annotation of over 2000 longevity-affecting genes (www.yeastgenome.org).

Yeast are widely used as a model for two different types of aging: replicative and chronological. Replicative lifespan (RLS) measures the number of daughter cells produced by a single mother cell before it reaches senescence. A distinguishing feature of RLS-aged cells is that they accumulate toxic extrachromosomal ribosomal DNA (rDNA) circles, controlled in large part by the NAD-dependent histone deacetylase Sir2, which functions to control recombination between rDNA repeat sequences [4–6]. Thus, understanding the precise mechanisms of protein behavior that contribute to RLS requires physical separation of mother from daughter cells in a growing culture, a process that necessarily makes discrete biochemical analysis relatively challenging.

In contrast to RLS, chronological lifespan (CLS) corresponds to the survival of cells beyond the point when they have stopped dividing. CLS assays in yeast measure survival decay after culture saturation and have become widely used as a simple model of post-mitotic and organismal longevity. In addition, cells harvested throughout the time course of an CLS experiment are easily subjected to protein biochemical analysis that is more difficult to achieve in RLS studies. Consequently, protein networks and pathways that control rates of chronological survival decay continue to emerge, providing ever increasing improvement in our understanding of the aging process.

In yeast, as in mammals, the PIK-type serine/threonine kinase Target of Rapamycin (TOR) is a master regulator of multiple pathways involved in CLS control. Yeast harbor two TOR kinases, Tor1 and Tor2, which are functional orthologs of mammalian TOR. Both kinases can be found in TORC1 protein complexes that function with Sch9 (ortholog of mammalian S6 kinase) to regulate autophagy – a CLS-positive process that enables survival of starving cells, as well as oxidative stress, and genomic instability – CLS-negative processes that promote cell death in chronologically aged cells [7]. Tor1 inhibits autophagosome (Atg1 complex) formation by direct multi-phosphorylation of the autophagosomal subunits resulting in disruption of Atg1 complex formation and inhibition of autophagy [8–10]. Sch9 is an AGC-type serine/threonine kinase that requires direct phosphorylation by Tor1 to enable its kinase activity, which is necessary for regulation of mitochondrial respiration, oxidative stress, as well as genome stability [11,12]. The combination of their multiple roles yields both *TOR1* and *SCH9* as negative regulators of CLS, and yeast lacking either gene exhibit increased longevity.

In addition to genetic factors, environment also plays a major role in the longevity of yeast as well as mammals. In general, calorie restriction (CR) is accompanied by reduced metabolic rate and consequential oxidative damage – factors that when left unchecked counteract long lifespan [13]. Indeed, evidence in support of this general phenomenon has been found in several model organisms including yeast, worms, flies, and rats [13]. Only more recently has evidence emerged that CR, without malnutrition, also extends the lifespan of monkeys and humans [14,15]. In this case, individuals experiencing CR generally exhibit classic yeast-like phenotypes as well as reduced prevalence of disease risk factors. Thus, mechanistic determinants of cellular lifespan are also critical factors for the survival of multicellular organisms, and studies at the cellular level remain a powerful tool for understanding the process.

While the precise mechanisms underlying aging in humans remain unclear, mammalian TOR (mTOR), which is well known to affect longevity in model organisms, is almost certainly involved. Indeed, long-standing evidence has shown that rapamycin, which increases autophagy in yeast [16], also extends the lifespan of mice and is also FDA approved for the treatment of a variety of diseases which otherwise reduce lifespan [17]. In many cases, the longevity effects of CR reflect a significant degree of protein and amino acid restriction – conditions that result in nitrogen starvation and deactivation of Tor [18–21]. Therefore, elucidating control mechanisms and pathways that participate in Tor function and longevity control is expected to be an important step in clarifying the precise mechanisms of aging and providing new targets for engineered lifespan-control.

We have used gene network analysis to identify genes with yet unknown function in chronological aging in the yeast model system. We discovered 19 genes with potential involvement in CLS, three of which were the mitogen activated protein kinases (MAPKs) *FUS3*, *KSS1*, and *HOG1*. In young cells, *HOG1* is essential for the high osmolarity/glycerol pathway, and has been shown previously to be a negative regulator of CLS in yeast as well as a genetic and physical interaction partner of Sch9 [22–24]. More recent evidence further suggests that basal activation of Hog1 is inhibited by rapamycin in *Candida albicans* by preventing Tor-mediated gene repression of two tyrosine phosphatases, *PTP2* and *PTP3* [25]. In contrast to Hog1, the CLS phenotypes of *FUS3* and *KSS1*, responsible for pheromone mating and nutrient starvation responses in yeast, are unknown. We therefore set out to analyze the importance of each MAPK in chronological aging, finding that Fus3, whose function is currently understood as restricted to the pheromone mating pathway of young cells, adopts non-canonical roles in aged cells that are important for regulating longevity.

## RESULTS

### Network-based identification of yeast MAPK's as aging genes

**Figure 1:**
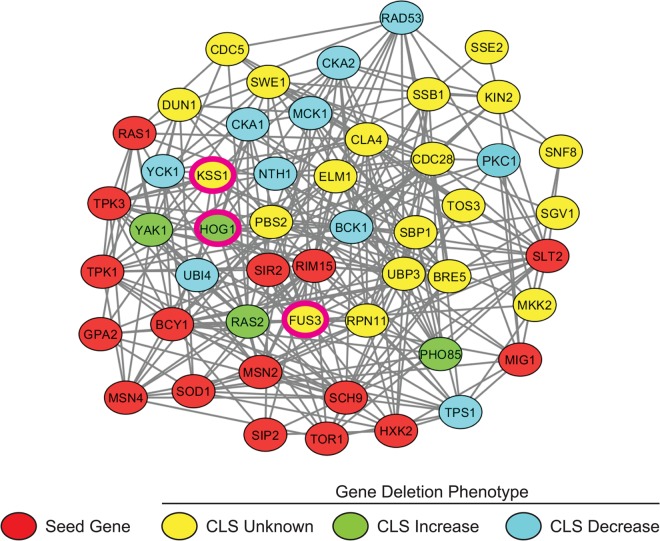
Subnetwork of yeast aging genes A subnetwork was extracted from the yeast whole genome network, YeastNetv2, using the network analysis tool GeNA. The network topology was displayed using Cytoscape and shows the top 50 genes/proteins highly associated with documented age-affecting seed genes. Seed genes (Red); CLS increased (Green); CLS decreased (Blue). MAPKs *FUS3*, *KSS1*, and *HOG1* are indicated by pink borders.

We assembled a set of 16 seed genes/proteins well known to be involved in the yeast aging process. Using the network analysis tool GeNA [26], a subnetwork of the top 50 genes highly co-expressed with the seed genes was then extracted from the yeast whole-genome network, YeastNet (see Materials and Methods) (Figure 1). Of the top 50 proteins in the network, 31 (including the 16 guide proteins) are known to be regulators of aging, while the remaining 19 were established as having no involvement in aging or their aging phenotype was not yet known (Table S1). Several of these proteins are necessary for a variety of signal transduction processes including the yeast mating response, osmotic stress response, nutrient response, cell wall stress response, as well as cell polarization pathways. Furthermore, the vast majority (31 proteins) of proteins in the subnetwork were either kinases or regulators of kinase activity. Three of these kinases were found to be mitogen-activated proteins kinases (MAPKs) – Fus3, Kss1, and Hog1 – kinases that regulate a wide variety of biological processes in yeast, and each of which is also highly conserved with its orthologous kinase in humans.

### Deletion of *FUS3*, *KSS1*, or *HOG1* MAPKs increase chronological lifespan

**Figure 2:**
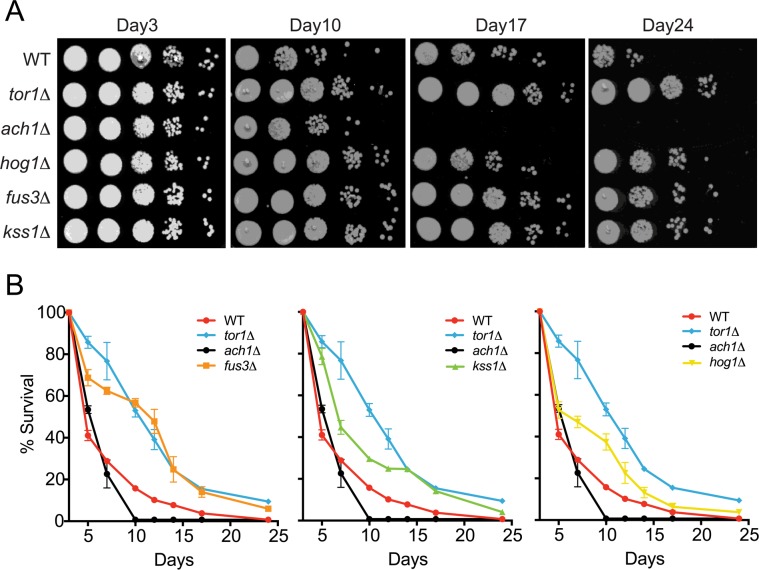
Cells lacking *FUS3* or *KSS1* exhibit elongated lifespan (**A**) Qualitative CLS was assayed for the indicated yeast strains at days 3, 10, 17, and 24 growing in SD medium with 2% glucose. 10-fold serial dilutions of each CLS culture were spotted (5 μl) onto rich media (YPD) agar plates and grown for 48 hours at 30°C followed by image capture on a flatbed scanner. (**B**) Quantitative CLS (qCLS) measurements from cultures treated as in A. Percent survival was calculated based on the outgrowth curves obtained from 24hr growth of cultures in microtiter plates (Materials and Methods). Error bars represent standard deviation across 3 analytical replicate experiments. For clarity, growth curves are separated to highlight *fus3Δ* (left); *kss1Δ* (middle); and *hog1Δ* (right) as well as controls for wild type lifespan (*WT*), increased (*tor1Δ*) and decreased (*ach1Δ*) lifespan.

Although the role of *HOG1* (ortholog of human p38 MAPK) in CLS has been shown previously by different laboratories [27,28], there is little to no published evidence in which the other MAPKs in yeast, *FUS3* and *KSS1* (orthologs of human *ERK2/ERK1*), had been similarly investigated. Indeed, the appearance of *FUS3* in our network analysis was unexpected since its sole function is to phosphorylate proteins that drive the yeast mating response – a process restricted to young cells (log-phase growth) activated by a pheromone stimulus [29]. Therefore, to determine whether *FUS3* or *KSS1* are also involved in yeast chronological aging, we performed a qualitative chronological lifespan (CLS) spotting assay with cells lacking either MAPK gene (*fus3Δ* or *kss1Δ*) grown in 2% glucose – a condition in which yeast cells are not nutrient stressed at the onset of the CLS experiment (Figure 2). Under these conditions, we found that cells lacking any one of the MAPKs (*fus3Δ*, *kss1Δ*, or *hog1Δ*; collectively referred to here as *mapkΔ*) exhibited a pronounced increase in longevity relative to wild type cells (measured as the degree of regrowth after each day of the aging experiment). In each case, the increase in survival was similar to that of *tor1Δ*, but unlike *ach1Δ* controls, for which longevity is well known to be increased or decreased, respectively (Figure 2A) [11, 30, 31].

To gain more quantitative insight, we monitored the survival of yeast using a quantitative CLS (qCLS) assay, in which cell population outgrowth is measured using a microplate reader. Unlike the spot assay, the qCLS assay enables the quantitative comparison of survival at each day during the experiment as well as the rate of decay (i.e. death rate) for the aging population [32,33]. The qCLS assay confirmed an increase in longevity of *mapkΔ* relative to wild type cells, each of which exhibited greater than 20% survival by the mid-point of the assay (day12) – a survival percentage 2 to 4-fold greater than that of wild type cells (Figure 2B). In comparison, *ach1Δ* cells died quickly and were completely dead by day12, while *tor1Δ* cells decayed gradually with ∼40% survival by day12. Strikingly, *fus3Δ* cells exhibited a survival decay profile very similar to that of *tor1Δ* cells (Figure 2B, left). In contrast, the decay in survival of *kss1Δ* and *hog1Δ* cells were less pronounced, suggesting a weaker overall effect on CLS compared to *fus3Δ* (Figure 2B, middle and right). By day24, survival of the wild type yeast was negligible, whereas all three *mapkΔ* strains remained viable. Consistent with earlier time points, the survival percentage of *fus3Δ* cells at day24 (6%) was very close to that of *tor1Δ* cells (9%) (Figure 2B). We conclude that deletion of individual MAPK genes (*FUS3* or *KSS1*), increases the longevity of yeast under normal growth conditions, with *fus3Δ* cells having the greatest effect compared to any other *mapkΔ*.

### Yeast lacking MAPKs exhibit increased starvation and stress tolerance

**Figure 3:**
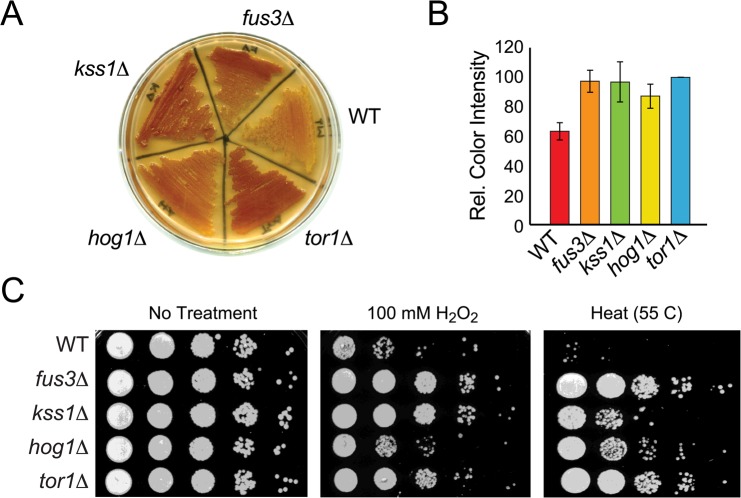
Cells lacking *FUS3* or *KSS1* exhibit increased stress resistance (**A**) Indicated yeast strains were patched onto YPD agar plates, grown for 2 days and then flooded with iodine to detect relative glycogen accumulation (observed as reddish-brown staining of the yeast patch; see Materials and Methods). A representative of three independent experiments is shown. (**B**) Quantitative comparison of glycogen accumulation shown in A. Images of replicate stained plates were quantified by densitometry. Error bars represent the standard deviation across 3 independent experiments. (**C**) Qualitative spot assay of 3-day old cultures grown in SD medium with 2% glucose (left panel), and exposed to 100 mM H_2_O_2_ for 30 min (middle panel) or to high temperature (55°C) for 10 min (right panel). Higher survival (growth) of the mutant strains relative to wild type *BY4742* (*WT*) indicates that cells lacking the indicated MAPKs exhibit different degrees of increased stress tolerance.

In aging cultures, nutrient deprivation leads to accumulation of the storage carbohydrate, glycogen, and yeast cells that accumulate glycogen are known to have a growth advantage compared to cells that do not [34,35]. Indeed, yeast mutations that extend lifespan have been shown to mimic a starvation state and accumulate glycogen even before entry into stationary phase [36–38]. To assess whether *mapkΔ* cells also mimic the starvation state, we measured glycogen iodine staining in each yeast strain. We observed significantly more glycogen iodine staining in *mapkΔ* as well as *tor1Δ* cells compared to wild type (Figure 3A). Based on image analysis for staining color intensity across three independent experiments, we found that *fus3Δ* and *kss1Δ* are similar to *tor1Δ* in their accumulation of glycogen, which is ∼1.5-fold greater than that of wild type cells and just slightly higher than that observed in *hog1Δ* cells (Figure 3A, B).

Since long-term survival of yeast is linked to the upregulation of various stress responses such as oxidative and heat stress [37,39], we also measured the survival of *mapkΔ* cells exposed to elevated temperature (55 C) or peroxide (H_2_O_2_) stress. Each *mapkΔ* strain, like *tor1Δ*, is more resistant to stress than wild type cells, although the degree of resistance is specific to which MAPK is deleted and which stress is applied. Specifically, *fus3Δ* cells, like *tor1Δ*, are insensitive to both heat and oxidative stress (Figure 3C). In contrast, *hog1Δ* cells are more resistant to heat than to oxidative stress, while *kss1Δ* cells are more resistant to oxidative than to heat stress. Thus, cells lacking any one MAPK exhibit starvation and stress phenotypes that are typical of long-lived mutant yeast such as *tor1Δ*.

### Yeast lacking *FUS3* exhibit distinctive lifespan response to carbon availability

**Figure 4:**
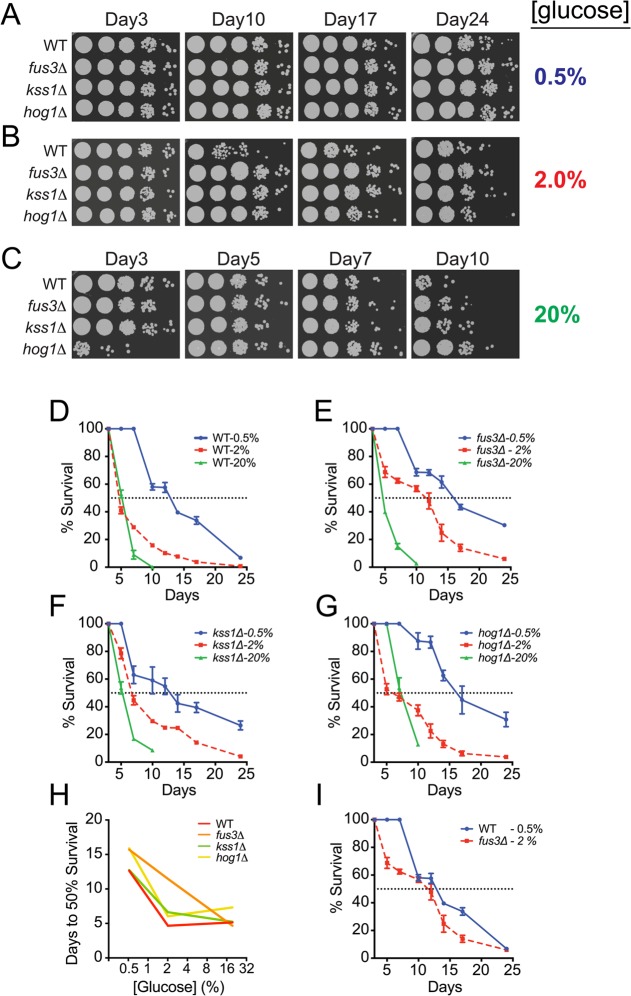
Yeast lacking *FUS3* exhibit sharp survival differences in response to fluctuating glucose levels (**A**) Qualitative CLS spot assay of indicated yeast strains grown in SD medium with 0.5% glucose. (**B**) Same as in A, but in SD medium with 2% glucose. (**C**) Same as in A but in SD medium with 20% glucose. (**D**-**G**) Overlaid survival decay profiles of *WT*, *fus3Δ*, *kss1Δ*, and *hog1Δ* grown in SD medium with 0.5%, 2%, or 20% glucose. (**H**) Plot of the number of days taken to reach 50% survival for the indicated yeast strains grown at different glucose concentrations, which shows that cells lacking *FUS3*, unlike other MAPKs, exhibit nearly linear sensitivity to fluctuations in glucose concentration. (**I**) Overlaid survival decay profiles of wild type cells grown under calorie restriction (0.5% glucose) and *fus3Δ* cells grown under calorie optimal (2% glucose) conditions, which reveals that deletion of *FUS3* has nearly similar longevity extension effect as does calorie restriction of wild type cells. Error bars throughout the figure represent the standard deviation across 3 analytical replicate experiments.

Availability and quality of carbon sources have a significant effect on the activity of proteins and pathways that play a role in aging [21,24], and calorie restriction, wherein the carbon and/or protein source is limiting, has been shown to increase lifespan in a variety of different eukaryotic organisms [40,41]. Moreover, the susceptibility of single gene deletion mutants to calorie restriction-induced lifespan elongation can be helpful in elucidating pathway-specific roles in nutrient utilization as cells progress in age [38]. Therefore, we examined CLS of *fus3Δ* and *kss1Δ* cells under calorie restricted (CR; 0.5% glucose) or calorie abundant (CA; 20% glucose) conditions. Under CR conditions, the longevity of wild type as well as each *mapkΔ* strain increased significantly with respect to the response of the same strain observed in optimal growth conditions (Figure 4A, B and S1A). In contrast, CA conditions dramatically decreased the longevity of all strains except for *hog1Δ*, which displayed moderate short-term resilience to high calorie growth medium – a phenotype that has also been observed for replicative aging of *hog1Δ* cells under CA conditions that is likely due to slower saturation rate induced by osmotic shock (Figure 4B, C and S1B) [27].

In further comparing the survival decay profiles of each strain with respect to glucose concentration, we discovered that fus3Δ, specifically, exhibits greater longevity than either kss1Δ or hog1Δ cells under normal growth conditions (2% glucose) (Figure 4D-G). By monitoring the number of days to 50% survival across all calorie conditions, the response of *fus3Δ* appears as a proportional increase in longevity with decreasing glucose concentration, suggesting that cells lacking the gene are less capable of homeostatic physiological control in response to fluctuating carbon availability (Figure 4H). In contrast, *kss1Δ* and *hog1Δ* cells exhibited very little difference in the number of days to 50% survival when comparing CR and optimal growth conditions, but a significant increase from normal to CA condition (Figure 4H). This was further reflected by the observation that *fus3Δ*, but not other cells, grown under optimal conditions (2% glucose) exhibit survival decay that is similar to wild type cells grown under CR conditions (Figure 4I). Taken together, these data demonstrate that cells lacking *FUS3* are distinct from cells lacking *KSS1* or *HOG1* and exhibit distinctive sensitivity to changes in calorie availability.

### Nitrogen starvation reverses the aging phenotypes of mapkΔ cells

**Figure 5:**
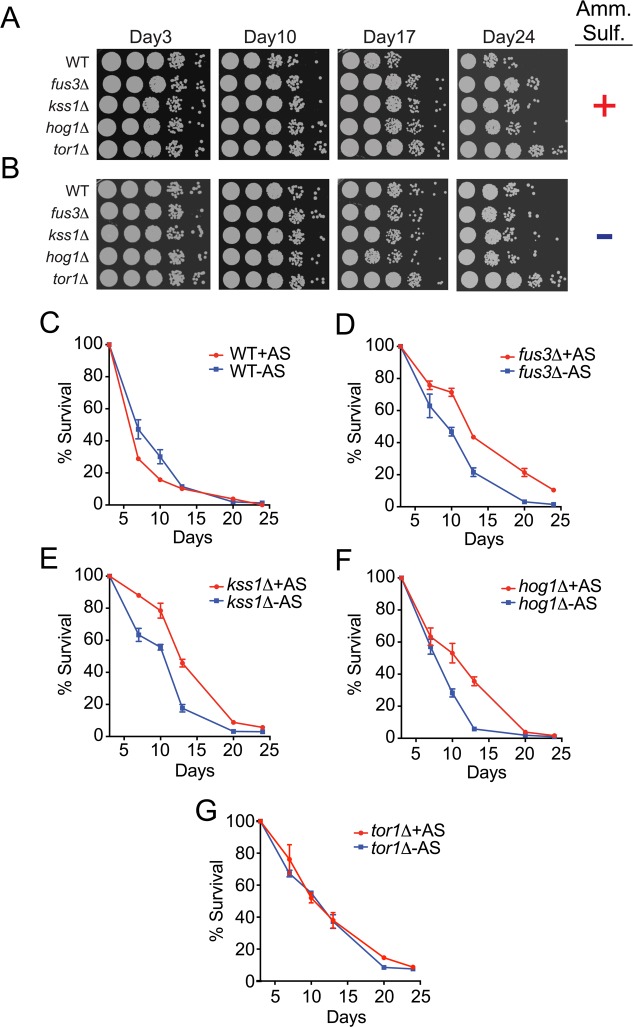
Yeast lacking any one *MAPK* exhibit a decrease in CLS upon nitrogen starvation (**A-B**) Qualitative CLS spot assay for the indicated genes grown in the presence (**A**) or absence (**B**) of ammonium sulfate. (**C-G**) qCLS curves comparing the survival decay of each *mapkΔ* strain and *tor1Δ* in the presence and absence of ammonium sulfate, representing nitrogen-optimal versus nitrogen-starved conditions, respectively.

Much like calorie restriction, limiting amino acids and other nitrogen sources, such as asparagine and ammonium sulfate, can also extend the longevity of yeast [21, 24, 37]. Therefore, we further examined the CLS phenotypes of individual *mapkΔ* strains in response to ammonium sulfate starvation. Consistent with previous reports, we observed a mild increase in the survival of wild type cells by both qualitative and quantitative assays (Figure 5A-C). Surprisingly however, deletion of *FUS3*, *KSS1* or *HOG1* resulted in decreased survival under nitrogen starved conditions – an opposite response compared to that of wild type cells (Figure 5A, B, D-F). In comparison, nitrogen starvation had no effect on *tor1Δ* cells (Figure 5G). When comparing results between strains, we found that the decrease in CLS for *mapkΔ* cells combined with the increase in CLS of wild type cells, largely diminished the differences in CLS of each *mapkΔ* strain (Figure S2A, B). This effect was most prominent for *hog1Δ* cells across all time points, but was only evident at older ages (day20) for *fus3Δ* and *kss1Δ* cells, at which point all *mapkΔ* cells exhibited survival percentages that were no different from wild type. We conclude that in the absence of each MAPK, cells are less capable of surviving in response to nitrogen starvation, and as a result exhibit little to no difference in CLS compared to wild type cells under such conditions. Thus, the longevity repressive effect of *FUS3*, *KSS1*, and *HOG1* in wild type cells grown under optimal conditions is largely nullified by the CLS-governing cellular state produced by nitrogen starvation.

### *FUS3* and *TOR1* are both required for short time-scale survival in the CLS experiment

Recent evidence suggests that *FUS3* is a positive regulator of autophagy in yeast and antagonistic to *TOR1*-dependent autophagy-repressing genes [42]. Tor1, a peripheral membrane-localized PIK-related protein kinase, is a “master regulator” of yeast nutritional control, especially as it pertains to nitrogen sensing and glucose signaling pathways [11, 19, 37]. Autophagy is repressed by active Tor1, but is derepressed when cells experience nitrogen starvation. Consequently, *tor1Δ* cells, which fail to repress autophagy, exhibit significantly greater survival percentages compared to wild type cells in the first days of a CLS experiment (Figure 2) [16,43]. Considering this and the fact that autophagy occurs early under normal growth conditions as evidenced by the fact that cell survival drops rapidly after day3 in the absence of autophagosome mutants [16], we hypothesized that *FUS3* would be important for driving the early-stage survival of *tor1Δ* cells in the CLS experiment.

**Figure 6:**
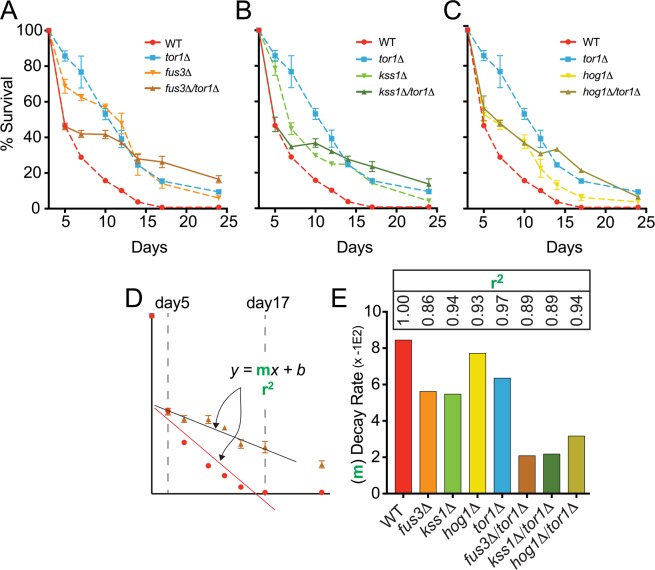
*FUS3* and *TOR1* interact genetically to control CLS (**A**-**C**) qCLS assay comparing the survival decay of *mapkΔ*/*tor1Δ* double deletion strains versus wild type and single gene deletion strains (overlaid from Figure 2, conducted on same plates). (**D**) Relative rates of survival decay from day5 to day17 were calculated using the slope value from linear regression analysis for each indicated strain. (**E**) The slope of the model (bars) and the coefficient of determination (r^2^) value representing the model fit (inset numbers) are shown to indicate the difference in relative decay rates of *WT*, single, and double gene deletion strains. Error bars throughout the figure represent the standard deviation across 3 analytical replicate experiments.

To test this hypothesis, we compared the CLS of *tor1Δ* and *mapkΔ*/*tor1Δ* cells using the qCLS assay. We found that the survival of *fus3Δ*/*tor1Δ* cells was distinctly lower than that of *fus3Δ* or *tor1Δ* cells within the first 5-7 days of the CLS experiment, mimicking the survival of wild type cells up to day5 of the experiment (Figure 6A). Beyond day5, *fus3Δ*/*tor1Δ* cells showed significantly elongated CLS that was even greater than that of *tor1Δ* cells by day24. Similar results were observed for *kss1Δ*/*tor1Δ* cells (Figure 6B). In contrast, *hog1Δ*/*tor1Δ* cells behaved much like *hog1Δ* cells (with the exception of day14 and day17), and exhibited lower CLS compared to *tor1Δ* cells by day24 (Figure 6C). Qualitative CLS measurements of double mutants, while not well resolved for early time points, showed an overall increase in CLS for all *mapkΔ*/*tor1Δ* strains over wild type and single mutant strains (Figure S3A, B), which we also confirmed using rapamycin to inhibit Tor1 in *mapkΔ* and wild type cells (Figure S3C).

In addition to altering the survival decay of *tor1Δ* cells in the early stages of the CLS experiment, we also noticed that the rate at which *mapkΔ*/*tor1Δ* cells decay beyond the early stage (i.e. beyond day5) was dramatically slower compared to single gene deletion strains. We quantified the relative decay rates by measuring the slope of the response (m) for each cell type between day5 and day17 (Figure 6D). We found that *fus3Δ*/*tor1Δ* and *kss1Δ*/*tor1Δ* cells exhibited a ∼2 to 3-fold reduction in survival decay rate compared to cells with either *MAPK* or *TOR1* genes deleted by themselves, and a ∼3 to 4-fold reduced rate compared to wild type cells (Figure 6E).

Taken together, these results show that loss of *FUS3* effectively nullifies the early-phase lifespan extension typically observed for *tor1Δ* cells. This data is consistent with a role of Fus3 as antagonistic to the role of Tor1 in autophagy that is known to occur early in the course of a CLS experiment. These results also suggest that the proposed role of Fus3 as a positive regulator of autophagy necessarily requires Tor1 since deletion of *FUS3* alone does not reduce the CLS of otherwise wild type cells.

### Yeast CLS is dependent upon phosphorylation-dependent activation of Fus3, Kss1, and Hog1

Fus3, Kss1, and Hog1 are each members of the canonical MAPK protein kinase family that includes Erk-type and p38 MAPKs found in higher eukaryotes [44]. As such, each kinase requires direct phosphorylation to activate its catalytic function [44]. Therefore, we asked whether the effect of Fus3 loss due to gene deletion was due simply to loss of the protein, which may be involved in protein interactions that facilitate CLS repression in wild type cells, or if Fus3 kinase activity was necessary to regulate CLS. To answer this question, we analyzed the CLS of MAPK activation-site mutants for Fus3^T180A,Y182F^ (*FAM*), as well as Kss1^T183A,Y185F^ (*KAM*) and Hog1^T174A,Y176F^ (*HAM*). These MAPK AM mutants are incapable of being activated by phosphorylation and are therefore catalytically-dead [44].

**Figure 7:**
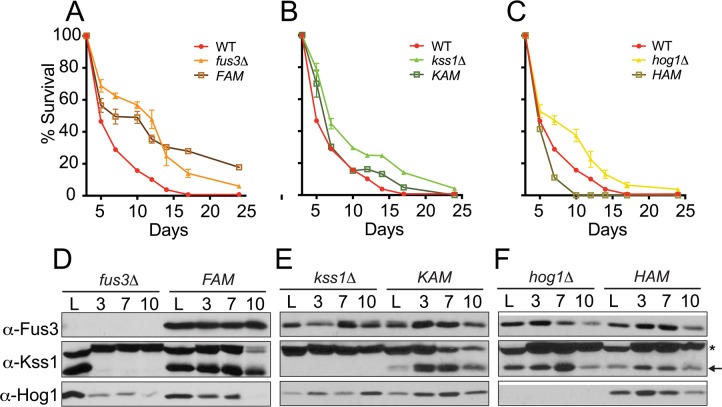
Cells expressing activation site mutant forms of Fus3 or Hog1 exhibit distinctive survival decay phenotypes (**A-C**) qCLS assay comparing the survival decay of MAPK activation site mutants versus single MAPK gene deletions (overlaid from Figure 2, conducted on same plates). Error bars throughout the figure represent the standard deviation across 3 analytical replicate experiments. (**D-F**) Western blot analysis of MAPK protein levels in cells harvested from experiments **A-C**. Activation site mutants are indicated as follows: *FAM*, Fus3^T180A,Y182F^; *KAM*, Kss1^T183A,Y185F^; and *HAM*, Hog1^T174A,Y176F^. Notably, Kss1 is absent from quiescent *fus3Δ* but not *FAM* cells. (^*^) A non-specific band above Kss1 is common to western blots with this antibody.

Results from the qCLS assay revealed dramatic distinctions in the survival decay profiles of each mutant. Indeed, each of the three activation mutant strains appear to have a distinct survival decay pheno-type that is unique in comparison to each other or to whole gene deletion mutants. Most strikingly, cells harboring *FAM* exhibit survival decay profiles that are distinctive from *fus3Δ* cells – much like the response of *fus3Δ*/*tor1Δ* cells (Figure 7A, S4A). Comparisons made between *KAM* or *HAM* and double mutants did not show this trend, or anything close to it, suggesting that it is specific to *FAM* cells (Figure S4B-D). Indeed, the survival of *KAM* cells was nearly identical to wild type cells (Figure 7B), while the survival decay in *HAM* cells, unlike *hog1Δ*, revealed a decrease in CLS compared to wild type cells (Figure 7C). Similar trends were also observed using the qualitative CLS assay (Figure S5). Thus, the kinase activity of Fus3 is important for maintaining normal longevity of wild type cells and preventing it results in a CLS response that mimics the loss of both TOR1 and FUS3, suggesting that Fus3 kinase activity is essential for the interplay between the two kinases early in the CLS response of yeast.

We also monitored each yeast strain for the presence of MAPK proteins by immunoblotting. We found that, in some cases, the protein stability of each MAPK behaved differently in response to deletion of other MAPKs. Most notably, Kss1 is quickly eliminated from *fus3Δ* cells between the initiation (log phase) and day3 (saturation) of the CLS experiment (Figure 7D). Thus, *fus3Δ* cells actually lack both Fus3 and Kss1 proteins. Surprisingly, we found that introducing activation-site mutations into Fus3 prevented this rapid loss of the MAPK (Figure 7D). We conclude that Fus3 kinase activity is necessary to maintain the stability of Kss1 at the onset of the chronological aging process. Furthermore, longevity phenotypes of *fus3Δ* cells necessarily reflect the loss of both Fus3 and Kss1 MAPKs at the protein level.

Introducing activation mutations did not appreciably alter the protein expression of Kss1 or Hog1, each of which exhibited relatively stable protein levels within the first 10 days of the assay (Figure 7E, F). Collectively, these data suggest that the inherent nature of MAPKs to be activatable by phosphorylation is critical to their role in CLS regulation in yeast – a counterintuitive observation for Fus3, for which activation is normally tightly restricted to mating pheromone pathway responses that are not expected during CLS experiments. Furthermore, the protein stability of MAPKs may, in some cases, be interdependent – an observation that is specifically evident for Kss1.

## DISCUSSION

### The unexpected involvement of mating pathway genes in yeast longevity

Analysis of gene or protein networks reconstructed from large-scale expression data or other data such as protein-protein interaction data, provide testable hypotheses for functional associations between genes/proteins involved in distinct biological processes [26, 45, 46]. We have analyzed the yeast whole genome network to determine genes/proteins functionally associated with the aging process – three of which correspond to the primary yeast MAPKs: Fus3, Kss1 and Hog1 (Figure 1). Since previous evidence has linked *HOG1* to lifespan control in yeast, this work serves primarily to identify the requirement for *FUS3* and *KSS1* in CLS – neither of which have been studied directly previously.

Functional roles for Hog1 in the chronological aging of yeast have been described previously, and its discovery in our aging gene network was therefore not surprising [24, 27, 28]. Previous results from these reports indicate both elongated as well as shortened longevity for cells that lack *HOG1*. In general, our results were consistent with a role for *HOG1* in restricting longevity of wild type cells. However, we also found that results achieved with *hog1Δ* cells were generally the most variable in comparison to *fus3Δ* or *kss1Δ* cells. Combined with the observation that *hog1Δ* cells exhibit survival decay profiles similar to that of wild type cells (in 2% glucose), this apparent contradiction in reported phenotypes may simply reflect stochastic fluctuation of *hog1Δ* longevity that is not significantly distinguishable from wild type cells.

The appearance of Kss1 in the aging gene network was also not surprising considering that its ortholog, Erk2, has been previously linked to chronological aging [47]. Within these studies, the authors also analyzed *kss1Δ* cells, finding that they were sensitive to heat and oxidative stress, and exhibited detectable but mild longevity defects compared to wild type cells when grown in the presence of excess amino acids. Under conditions in which excess amino acids are not supplemented in the culture medium, we find that cells lacking *KSS1* have marked sensitivity to heat stress, are largely insensitive to oxidative stress, and have a moderate increase in longevity. These conclusions are supported by evidence from over five different independent experiments, all of which resulted in similar effects that occasionally varied only due to relative fluctuations in the wild type response (data not shown).

In contrast to Kss1 and Hog1, our discovery of Fus3 in the aging gene network was least expected. Indeed, longstanding evidence has reported extensively on the role of Fus3 in the pheromone mating pathway, which is activated exclusively in response to exogenous peptide mating pheromones that activate G protein coupled receptors on the surface of young cells [48,49]. Therefore, the observation that activation sites in Fus3 are necessary to facilitate its role in chronological longevity is unexpected. Thus, systems-level network analyses, which enable unbiased gene clustering, have revealed unexpected roles of MAPKs that would not be determined by intuition alone.

In addition to MAPKs, several other genes with unknown roles in chronological aging were also identified by gene network analysis and within our conservative list of 50 genes (Figure 1, yellow circles). Surprisingly, many of these genes are directly linked to the pheromone mating pathway that activates Fus3 and Kss1, and are consistent with a role for MAPKs in chronological aging. For example, several genes (*ELM1*, *CLA4*, *TOS3*, *MKK2*, *PBS2*, *BRE5*) correspond to kinases or other proteins involved in MAPK activation, signaling, or regulation – many of which have been shown to modulate Fus3 and Kss1, or Hog1 activity in young cells exposed to mating pheromone or osmotic stress, respectively (Table S1). Other genes (*CDC28*, *SWE1*, *CDC5*, *SGV1*, *DUN1*) are directly involved in cell cycle checkpoint or progression mechanisms that are critical for cell division and so are also likely required for longevity. While a more liberal threshold would likely identify several more putative aging genes, we've demonstrated here that MAPKs and other proteins involved in yeast mating are contained in a conservatively-thresholded aging gene network.

### Yeast lacking individual MAPKs exhibit several hallmarks of long-lived yeast

In addition to elongated lifespan, we have confirmed that *fus3Δ* and *kss1Δ* share additional phenotypes commonly observed for well-characterized long-lived yeast genotypes (Figure 3). Indeed, previous studies have shown that starvation tolerance and stress resistance are often hallmarks of long-lived mutants [37, 50–52]. The ability to accumulate the storage carbohydrate glycogen before the starvation period, or to reduce the accumulation of reactive oxygen species through expression of stress resistance proteins is thought to explain the reason for such hallmarks. Our studies show that *fus3Δ, kss1Δ,* and *hog1Δ* strains not only accumulate higher amounts of glycogen in their cells, but also exhibit enhanced resistance to both oxidative and heat stress, when compared to wild type cells. In contrast, previous studies have shown sensitivity of *hog1Δ* cells to oxidative and heat stress [53–55]. The discrepancy in these results could be due to the difference in the growth or experimental conditions and/or the yeast strains used in the previous studies. For example, the heat shock studies performed by Winkler et al. used a different mating type yeast and 22 hours of heat shock at 39°C versus 10 minutes at 55°C used in our studies. Indeed, we have found in several experiments shown here that time is a critical factor that should be considered when studying the comparative effects of mutation on CLS. Nevertheless, our data support existing evidence that starvation tolerance as well as stress resistance likely play a role in enhancing the longevity of *mapkΔ* strains.

### Evidence supporting a model for antagonistic control of autophagy by *FUS3* and *TOR1*

Under optimal growth conditions, the rapamycin-sensitive TORC1 complex (which harbors Tor1 as a catalytic subunit) controls several aspects of cell growth and proliferation, and also inhibits autophagy and the stress response [24,56]. Cells lacking *TOR1* are also stress tolerant and incapable of inhibiting autophagy – factors that enable longer lifespan [37,43]. Tor1 is also a nitrogen sensor and is activated in the presence of excess nitrogen sources (such as ammonium ion), but inhibited under nitrogen starvation conditions [19–21]. We have shown that yeast lacking *FUS3*, in particular, mimic the behavior of cells lacking *TOR1* in several ways: increased longevity, increased storage of glycogen, and increased tolerance to nutrient, oxidative, or temperature stress (Figures 2–3). However, unlike *tor1Δ* cells, in which the CLS response is insensitive to nitrogen starvation (Figure 5G) [28], the CLS of *fus3Δ* and the other *mapkΔ* cells is shortened under the stress (Figure 5D-F).

The mechanism underlying the sensitivity of yeast to nitrogen starvation in *mapkΔ* cells, such as *fus3Δ*, is unknown. However, clues about the mechanism may lie in the relationship of MAPKs to autophagy – a process that is required to promote lifespan at early stages of aging in yeast [16]. Indeed, recent evidence suggests that *FUS3* is necessary to promote autophagy, and that *fus3Δ* cells are significantly less efficient at the process compared to wild type cells [42]. In contrast, Tor1 inhibits autophagy through several mechanisms including: one, direct phosphorylation of Sch9, which functions in parallel with PKA to inhibit Rim15 kinase and transcription factors Msn2/4 [12,57]; two, direct multi-phosphorylation of the autophagosomal subunit, Atg13, which results in disruption of autophagosome complex formation [8–10]; and three, through activation of protein phosphatase 2A – a negative regulator of autophagy that also prevents stress-dependent activation of Hog1 [25,58]. Many of our experimental results are consistent with the idea that Fus3 is antagonistic to Tor1 and promotes autophagy. First, the survival of *fus3Δ* cells is significantly reduced under nitrogen starvation, a condition that normally promotes autophagy. Indeed, in the absence of ammonium sulfate, the CLS response of *fus3Δ* cells approaches that of wild type cells and is shorter than that of *tor1Δ* cells (Figure S2B). In the presence of ammonium sulfate, *fus3Δ* cells exhibit CLS that is longer than that of *tor1Δ* cells, further suggesting that Fus3 is somehow regulated in a nitrogen/ammonium-dependent manner that is yet unknown (Figure S2A). Second, when Tor1-dependent autophagy repression is synthetically disabled (by deletion of *TOR1*), the simultaneous absence of Fus3 (i.e. *fus3Δ*/*tor1Δ*) nullifies the CLS extension typically observed in the early stages (up to day5) of the qCLS assay when Fus3 is present (i.e. *tor1Δ*) (Figure 6A).

Third, exclusive expression of non-activatable, kinase-dead Fus3 (i.e. *FAM*) also mimics the behavior of *fus3Δ*/*tor1Δ* cells wherein survival decay is rapid up to day5 (Figure 7A).

**Figure 8:**
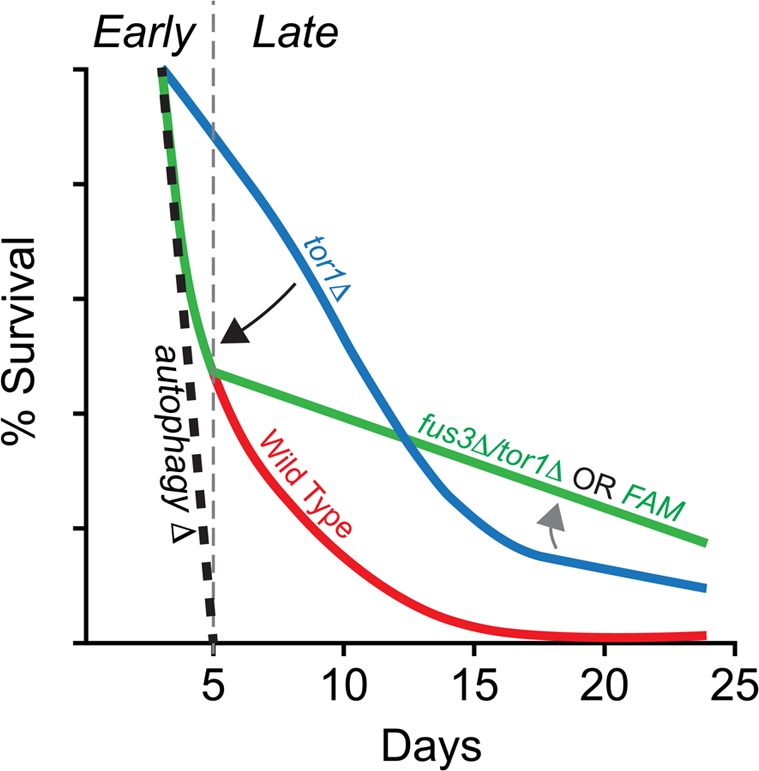
Summary of the combined effects of *FUS3* and *TOR1* on CLS in yeast (**A**) Diagram indicating the dynamic CLS response of *mapkΔ* and *tor1Δ* cells. The elongated lifespan of *tor1Δ* cells is dependent on the ability of cells to carry out autophagocytosis, since cells lacking a functional autophagosome (e.g. *atg1Δ*) die rapidly, regardless of the functional activity (or presence) of Tor1 in cells. Thus, the elongated lifespan phenotype of *tor1Δ* cells can be nullified by autophagy-null mutations (e.g. *atg1Δ*; black dashed line) (taken from Alvers et al. [16]). Early in the CLS experiment, deletion of *FUS3* (which is required for efficient autophagy [42]) has a similar effect by reducing the survival of *tor1Δ* yeast (*fus3Δ*/*tor1Δ*; black arrow). This effect is similar to the decay rate of wild type cells within the first week of the CLS experiment, which may or may not be coincidental. The effect is also not as extreme as is observed for autophagy nullification. In later stages of the CLS assay, *fus3Δ*/*tor1Δ* cells exhibit considerable change in decay rate, indicating a possible transition to another stage in which the balance between antagonistic autophagy regulators (as well as other CLS-controlling processes) shifts (grey arrow) in a manner that is not normally observed when both genes are present in wild type form. This two-stage response likely requires the ability of Fus3 to be activated by phosphorylation since substitution of Fus3 with Fus3^T180A,Y182F^ (i.e. *FAM*) exhibits the same CLS response as deletion of both *FUS3* and *TOR1* together.

Taken together, our data provide supporting evidence of a genetic interaction between *FUS3* and *TOR1* that may rely on the kinase activity of Fus3. We speculatively propose that this interaction is centered on the autophagy response. Our evidence to support this proposed mechanism come from the fact that *FUS3* is required for optimal levels of autophagy [16], that deletion of *FUS3* in a *tor1Δ* background nullifies the typical survival response of *tor1Δ* cells early in the CLS assay, and that inactivation of Fus3 kinase activity results in the same effect (this work) (Figure 8). This idea requires an assumption that the aging “program” is dynamic, perhaps even stage-like, such that pathways and survival “programs” at the early stages of aging are different from those at later stages. While this does not appear to be a commonly discussed aspect of chronological aging, distinct stages have been identified for the replicative aging process (reviewed in [59]). Within this paradigm, autophagy is a process that must occur at the earliest of stages. Indeed, both *BY4741* and *BY4742* yeast that lack core autophagosomal subunits die within a matter of ∼1 week in a typical CLS experiment [16, 60].

Interestingly, the survival of *tor1Δ* cells at early stages is completely dependent on this early autophagy response since inhibition of the kinase with rapamycin in autophagy-deficient cells (e.g. *atg1Δ*) nullifies typical *tor1Δ* survival at early time points [16]. We find that deletion of *FUS3* in *tor1Δ* cells has a similar yet less potent effect on survival compared to autophagosomal mutants (based on data from Alvers et al. 2009), resulting in early stage survival decay that is closer to the wild type than the *tor1Δ* response within the first 5-7 days of sampling (Figure 6A, S6). This CLS response of *fus3Δ*/*tor1Δ* cells suggests that *FUS3* could be one of multiple factors that are necessary to promote autophagy and that nullifying its effect (by deletion of *FUS3* or activation mutation) could be a consequence of its known positive impact on autophagy [42]. Indeed, these data would suggest a potential competitive molecular effort to restrain (*TOR1*) and promote (*FUS3* and other genes) autophagy during the early stage of chronological aging. Beyond day5, the dramatic reduction in decay rate of *fus3Δ*/*tor1Δ* or *FAM* cells suggests that the transition into another stage of the aging program may take place. While we cannot explain exactly why this change occurs presently, we can say that it is impacted by the function of MAPKs and Tor1.

In addition to providing evidence in support of *FUS3* as a positive regulator of autophagy, we show that deletion of *FUS3* alone increases cell survival under normal growth conditions. This apparent contradiction in light of the *fus3Δ*/*tor1Δ* results suggests that the autophagy-promoting function of Fus3 requires Tor1 and is in fact further evidence that Fus3 and Tor1 are both required to achieve wild type CLS.

### How can Fus3 activation, which is necessary for normal CLS, occur in the absence of mating pheromone?

Unlike Kss1 and Hog1, which have established roles in stress response, the established primary role of Fus3 is to facilitate the mating response – a process that is tightly restricted from occurring under stress conditions. Indeed, activation of the kinase by dual phosphorylation requires direct interaction with a MAPK scaffold protein (Ste5) and an upstream MAPK-kinase (Ste7), which are inhibited in the absence of mating pheromone [61–63]. Therefore, illuminating potential roles for Fus3 and its kinase activity in chronological aging, and in the absence of pheromone, is an important hypothesis generated by this work. The evidence we provide here suggests an emerging role for the kinase-active form of Fus3 in the process of autophagy and chronological lifespan. Specifically, we find that cells incapable of activating the catalytic activity of Fus3 (*FAM*) exhibit a survival decay response identical to cells that lack both Fus3 and Tor1 (*fus3Δ*/*tor1Δ*) (Figure S4A,D). Furthermore, comparisons made for activation mutant forms of Kss1 and Hog1 do not show this pattern, or anything close to it, suggesting that it is specific to *FAM* cells (Figure S4B-D). Thus, the kinase activity of Fus3 is important for the genetic interaction observed between *TOR1* and *FUS3*, autophagy, and chronological longevity.

In addition to Fus3, we have also demonstrated the role of Kss1 and Hog1 activation in chronological aging. Cells lacking the activation mutant form of Kss1 (*KAM*) exhibit survival decay rates that are nearly identical to wild type cells, whereas complete deletion of the kinase gene results in elongated longevity (Figure 7B). These data suggest that Kss1 protein, but not its kinase activity, plays a role in regulating lifespan. In contrast, cells lacking the activation mutant form of Hog1 (*HAM*) show an equal and opposite effect on longevity compared to complete deletion of the kinase gene (Figure 7C). Thus, these data suggest that Hog1 kinase activity is essential for maintaining longevity in wild type cells, a result that is consistent with previous studies implicating Hog1 in the Tor1 pathway [24, 25, 64, 65].

Three observations suggest that activation mutant CLS phenotypes result from failure to activate the kinase rather than disruption in kinase structure and function. First, the CLS response of each activation mutant is distinct from that of whole gene deletion strains, which would not be expected if mutation of these highly conserved residues disrupted the fold structure of the kinase. Second, immunoblotting indicates that MAPKs with activation site mutations are stably expressed, which would not be expected if the mutations were destabilizing to the protein fold structure. Indeed, identical activation site mutations are used routinely for studying the impact of activation of each of these MAPKs, in which cases catalytic activity is lost but protein interactions are retained. Third, using phos-tag gels to identify the mono and di-phosphorylated states of each MAPK in log phase cells, we observed that Fus3 was basally mono and di-phosphorylated at the onset of the aging experiment (Figure S7). Both mono and di-phosphorylation was robust in comparison to basal phosphorylation in *BY4741* yeast, though still much lower than that of pheromone-stimulated *BY4741* yeast. Hog1, in which activation mutation results in a CLS phenotype that is opposite to *FAM*, was not detected in the mono or di-phosphorylated state in log phase cells, suggesting that activation of the MAPK may only happen later in the aging process when stress levels have increased significantly. While regulation mechanisms for basal MAPK phosphorylation are known for Fus3 in young cells [61–63], they may or may not explain the mechanisms that regulate MAPKs during the aging process. Understanding the mechanisms underlying basal activation of Fus3 in aging cells will require further analysis at the molecular level and is certainly warranted in light of this data.

Broadly, these results indicate that the paradigms defining protein relationships of MAPK signaling pathways in young cells may have to be considered again in the context of aged cells. We have accessed unexpected relationships between mating-specific MAPKs and proteins that effect CLS in yeast using gene network analysis methods. The data suggest that important age-specific protein networks, while not obvious in young cells, may be elucidated by considering systems level rather than pathway level connectivity. Our work highlights MAPKs involved in the mating response of yeast, namely Fus3 and Kss1, as important early-stage regulators of the CLS program. This represents only the third and fourth genes from the yeast mating pathway that have been studied for effects on CLS (the others are Gpa1 and Ste50). Indeed, based on our survey of the literature and the *Saccharomyces cerevisiae* genome database, most mating pathway genes have not been studied for CLS effects in *BY4742* previously. What data does exist is found in *BY4741* for which several mating pathway genes have been tested in a large-scale microarray screen for chronological aging factors (e.g. *STE2, STE3, SST2, GPA1, STE4, FAR1, STE20, STE11, STE50, STE7*) [60]. When parsing these microarray results for genes expected to reduce or prevent Fus3 activation when deleted (*STE2, STE3, FAR1, STE4, STE11, STE50, STE7*), versus genes that should have a positive or neutral impact on Fus3 activation (*GPA1, SST2*), we found about 65% positive correlation (at day9 of their study) and about 47% positive correlation (at day33 of their study), suggesting that the effects we observe for Fus3 activation-dependent CLS control are not easily predictable based on canonical mating pathway architecture [60].

However, the lack of a wild type control in the supplemental microarray data combined with the fact that the experiments were not intended for high-resolution time point analysis, makes it difficult to be certain of the CLS effects of mating pathway genes, which were not the focus of their work. Considering that several mating pathway components are degraded in response to cell fusion [66], or under conditions of high cell density (Torres, unpublished data), it will be important first to understand how “complete” the mating pathway is in aged cells, and whether components that are retained during chronological aging are important in maintaining normal CLS.

## MATERIALS AND METHODS

### Yeast strains and genetic manipulations

All strains used in this study were derived from *BY4742* background (Table 1). Wild type and single gene deletion mutants of *BY4742* (*MATα his3Δ1 leu2Δ0 lys2Δ0 ura3Δ0*) were purchased from GE Dharmacon (Lafayette, CO). These were subsequently used to generate other mutants of interest by PCR-mediated gene disruption and/or delitto perfetto [67]. Yeast transformations were performed using standard methods [68], and all gene disruptions were verified by either PCR and/or DNA sequencing. Cultures were grown in standard YPD medium or synthetic defined (SD) medium with 2% glucose at 30°C as described previously, unless otherwise stated [32,69]. Double mutant strains were verified by immunoblotting to confirm the absence of the respective MAPK and Tor1 in each strain (Figure S3A).

### Yeast whole genome network analysis

The Yeast whole genome network (YeastNet v2) was downloaded from (http://www.inetbio.org/yeastnet/). YeastNet is a probabilistic functional network comprising of ∼5,483 yeast proteins, and reconstructed using various large scale datasets (microarray, genetic and physical interactions), as well as low throughput published studies [45,46]. Network analysis was performed using GeNA and visualized in Cytoscape [26,70]. GeNA takes seed/guide genes as input and ranks genes from the whole genome network based on their significance of interactions and relevance to the whole set of seed genes. Taking the top 50 such ranked genes, we extracted an aging gene subnetwork. BioGRID database was further utilized to assess protein-protein interactions amongst these 50 genes [71].

### Chronological lifespan assay

Yeast chronological lifespan (CLS) assays were done as previously described [27,69]. Briefly, fresh cultures from single colonies were grown overnight in YPD medium, diluted to an OD_660_ of 0.2 and grown to OD_660_ ∼0.8 – 1. The log phase cultures were then diluted in water to 1 × 10^6^ cells/mL and stored at 4°C for 1-2 days. Aging assays were initiated with 1 × 10^4^ cells/mL for all different genotypes in 4 mL SD medium, and maintained at 30°C with shaking at 230 rpm for the entire duration of the experiment [69]. Samples were taken at regular intervals starting on Day 3. Serial 10-fold dilutions of each sample were then plated onto YPD plates, and growth patterns for each genotype were recorded after 2 days of incubation at 30°C. For determining the effect of nitrogen, and glucose on lifespan, cultures were grown in SD medium without ammonium sulfate, or with varying concentrations of glucose (0.5%, 2% and 20%). High-throughput plate screening was achieved as previously described [69]. For each interval, 5 μL of the 10^−2^ diluted sample was added to 100 μL of YPD medium, and cell growth was monitored by absorbance (OD_660_) every 10 minutes for 24 hrs using a Synergy HT microplate reader (BioTek, Winooski, VT). Sampling error was estimated by triplicate analysis of each strain and each experiment was repeated at least 3 independent times.

### Glycogen accumulation

Glycogen accumulation was measured as described previously [72]. Yeast strains were grown on YPD agar plates for 2-3 days at 30°C. The plates were then flooded with 6 mL of freshly made iodine solution (1 mg/mL I_2_ in 10 mg/mL KI) for 1-2 min. Images were scanned and color density was measured using ImageJ software [73].

### Heat and oxidative stress resistance

Yeast cultures were started in SD medium as per the CLS assay. Heat stress assays were performed with cells grown in SD medium for 3 days (stationary phase). For determining heat stress resistance, yeast strains were exposed to either 30°C (control) or 55°C (heat shock) for 10 min, and immediately cooled on ice for 1-2 min. Serial 10-fold dilutions of control and heat-shocked cultures were plated onto YPD plates and allowed to grow for 2 days at 30°C. The oxidative stress assay was performed by treating 3-day old cells with 100mM H_2_O_2_ for 30 min at 30°C. Treated and control cells were spotted onto YPD plates, and results were recorded two days after growth at 30°C.

### Inhibition of the TOR pathway

A stock solution of rapamycin (ApexBio; Houston, TX) was made in 90% ethanol and 10% Tween-20 at 1 mg/mL concentration, and stored at −20°C. Yeast cultures were started in SD medium as per the aging assay. Rapamycin at 4 ng/mL final concentration (treated cells) or the drug vehicle (control) was added after 6 hrs of growth at 30°C. Cultures were maintained at 30°C for the entire duration of the experiment and samples were taken out at regular intervals. Serial dilutions and spotting of yeast cultures were performed as per the CLS assay.

### Protein extraction and immunoblot analysis

Whole cell protein extracts from wild-type *BY4742* and mutants were prepared from cultures grown in SD medium. Cell pellets from samples taken at various time-points were harvested by centrifugation, and protein extraction in trichloroacetic acid as described previously [74]. For immunoblot analyses, approximately 50μg protein was electrophoresed through 7.5% SDS polyacrylamide gels and transferred onto nitrocellulose membranes (Whatman, Kent, UK) as previously described [75]. Membranes were probed with rabbit polyclonal or mouse monoclonal antibodies to Fus3 (1:350; SC-6773), Kss1 (1:1250; SC-6775-R), Hog1 (1:350; SC-6815), and Tor1 (1:200; SC-11900) (Santa Cruz Biotechnologies; Dallas, TX). Target proteins were visualized using goat and rabbit secondary antibodies conjugated to horse-radish peroxidase followed by ECL detection (Pierce, Erembodegum, Belgium) as described previously [76].

## SUPPLEMENTARY MATERIAL


