# Supplementary material for: Mitogen-activated protein kinases, Fus3 and Kss1, regulate chronological lifespan in yeast
Source: Aging (Albany NY). 2017 Dec 21;9(12):2587–609. doi: 10.18632/aging.101350 (PMC5764394; doi:10.18632/aging.101350)
Supplement: Supplementary file 1 [file aging-09-2587-s001.pdf]

SUPPLEMENTARY MATERIAL

Table S1. Table of putative longevity genes discovered in this study. The description and longevity phenotype of genes highly associated with the aging seed genes (as shown in Figure 1) is shown here. The known aging phenotype of the null mutants for each respective gene was retrieved from SGD (http://www.yeastgenome.org/).

GENE	DESCRIPTION	PROTEIN TYPE	LINK TO PHEROMONE PATHWAY?	HUMAN ORTHOLOG
ELM1	morphogenesis checkpoint kinase (one of 3 SNF activating kinases)	Kinase	YES	calcium/calmodulin-dependent protein kinase kinase; sreine/threonine kinase 11
CLA4	Cdc42-activated signal transducing kinase (Ste20-like)	kinase	YES	oxidative stress responsive 1; p21 protein (Cdc42/Rac)-activated kinase
TOS3	Ortholog of human LKB1 and one of 3 SNF activating kinases)	Kinase	YES	calcium/calmodulin-dependent protein kinase kinase
SNF8	component of ESCRT-II complex for ubiquitni-dependent sorting			SNF8, ESCRT-II complex subunit
PKC1	kinase essential for cell wall remodeling and integrity	kinase	YES	AKT1 - v-akt murine thymoma viral oncogene homolog
PBS2	MAPKK	kinase	YES	mitogen-activated protein kinase kinase 1-7
CDC28	master cyclin dependent kinase	kinase		CDK1 - cyclin dependent kinase 1
BRE5	ubiquitin protease cofactor (Ubp3)		YES	GTPase activating protein (SH3 domain) binding protein 1
SWE1	cyclin-dependent kinase (G2/M); negative regulator of Cdc28	kinase		WEE1 G2 checkpoint kinase
CDC5	polo-like kinase involved in cell cycle	kinase		Polo-like kinase
KIN2	regulates exocytosis	kinase		Hormonally up-regulated Neu-associated kinase
SGV1	cyclin-dependent kinase (G2/M)	kinase		CDK9 - cyclin dependent kinase 9
SBP1	elongation factor binding protein			none
DUN1	DNA damage checkpoint	kinase		CHEK2 - checkpoint kinase 2; calcium/calmodulin-dependent protein kinase 1
MKK2	PKC pathway MAPKK	kinase	YES	mitogen-activated protein kinase kinase 1/2
SSE2	Stress seventy subfamily E; heat shock protein	HSP		HSPA4 - heat shock 70kDa protein 4

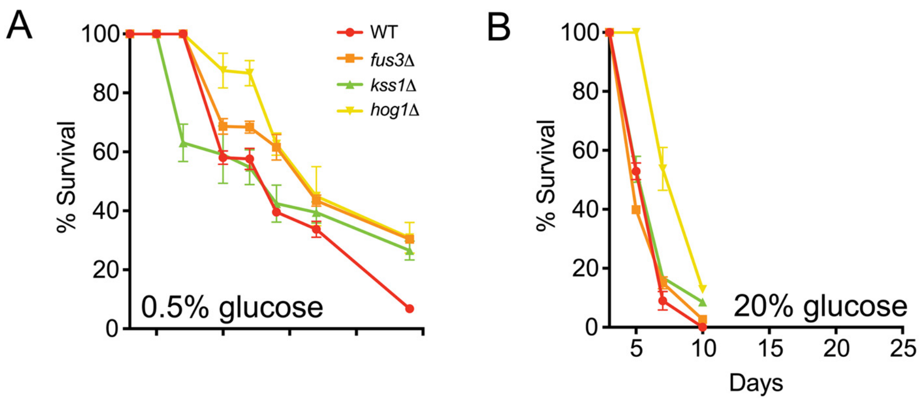

Figure S1. Yeast lacking *FUS3* are distinctly sensitive to fluctuations in calorie availability. (**A**) qCLS curves showing percent survival of the indicated yeast strains grown in SD medium with 0.5% glucose (calorie restricted). (**B**) Same as in **A** but in SD medium with 20% glucose (calorie abundant). Dashed line indicates that the *hog1Δ* culture did not reach saturation by day 3. Error bars throughout the figure represent the standard deviation across 3 analytical replicate experiments.

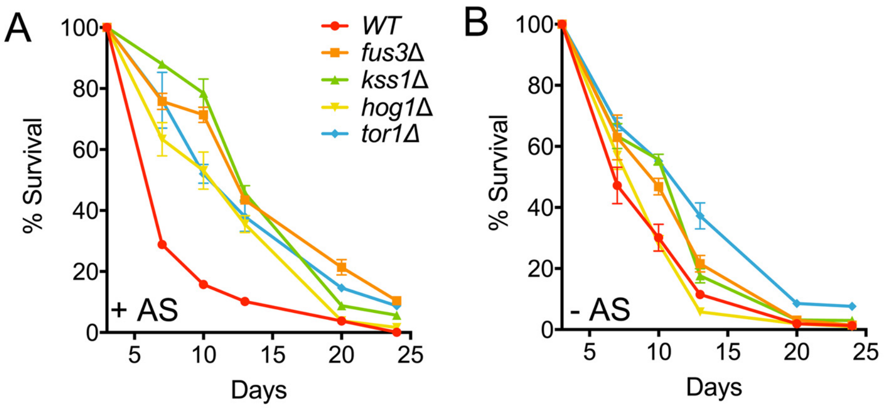

Figure S2. Yeast lacking specific MAPK genes exhibit reversal of the wild type longevity response to nitrogen starvation. (**A**) qCLS curves showing percent survival of the indicated yeast strains grown in SD medium with ammonium sulfate (+AS; nitrogen optimal). (**B**) Same as in **A** but in SD medium without ammonium sulfate (-AS; nitrogen starved). Error bars throughout the figure represent the standard deviation across 3 analytical replicate experiments.

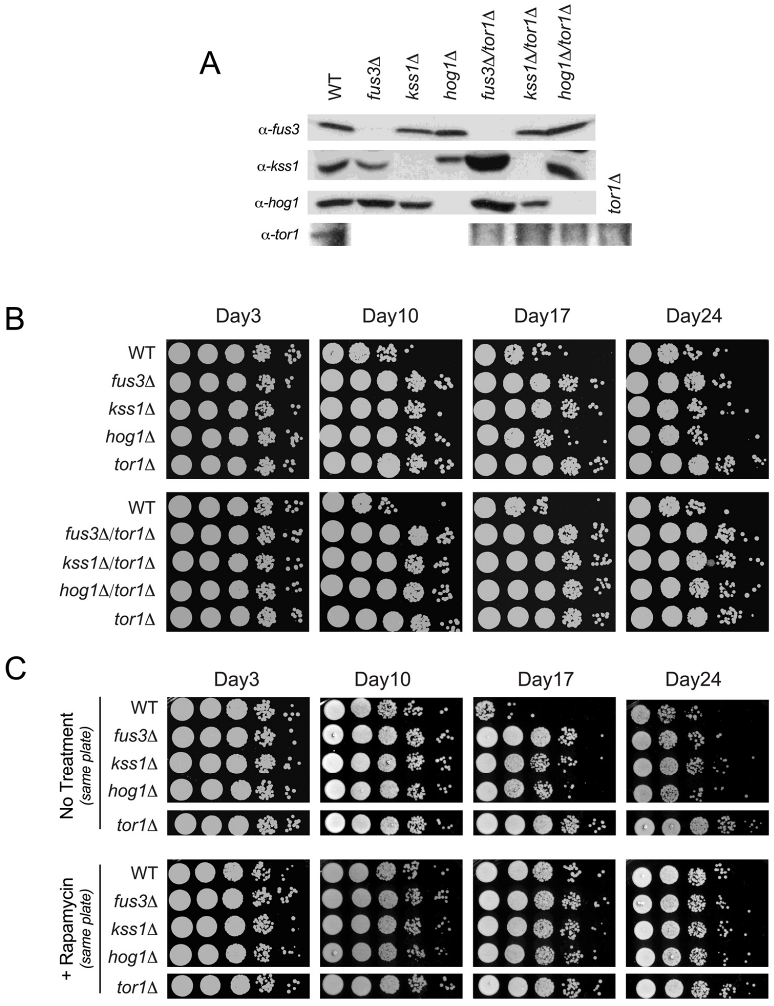

Figure S3. Qualitative CLS results for *mapkΔ* cells in which Tor1 is inhibited or deleted. (**A**) Equal amounts of total protein extracted from log phase cultures of *BY4742* and derivative strains grown in YPD medium separated by 7.5% SDS-PAGE followed by transfer to nitrocellulose membranes. Protein-loaded membranes were probed with antibodies to Fus3, Kss1, Hog1, and Tor1 as described in materials and methods. (**B**) Qualitative CLS spot assay of yeast strains lacking the indicated single genes (top), versus those lacking both *MAPK* and *TOR1* genes together (bottom). (**C**) Qualitative CLS spot assay of the indicated yeast strains in the presence (bottom) or absence (top) of Rapamycin (4 ng/ml), showing that inhibition of Tor1 in *mapkΔ* strains mimics inhibition of Tor1 in wild type (*WT*) cells.

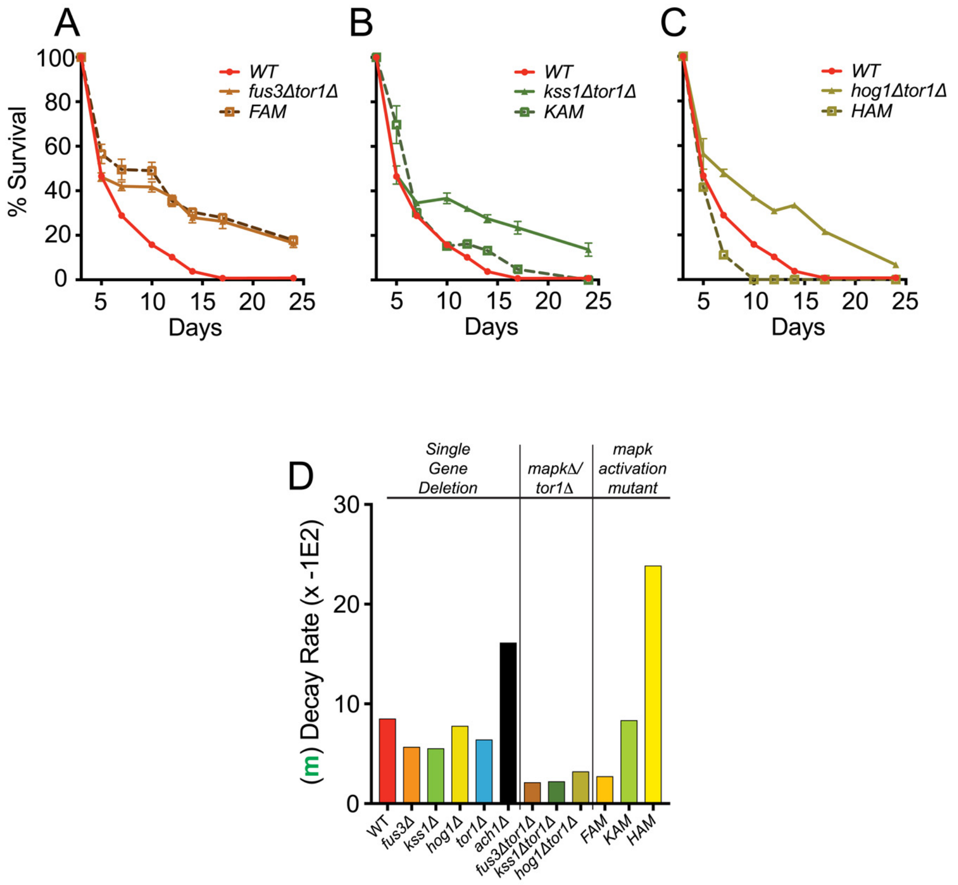

Figure S4. Survival decay of yeast harboring non-activatable Fus3 mimics that of yeast lacking both *FUS3* and *TOR1*. (**A**-**C**) Overlaid survival decay profiles of *mapkΔ*/*tor1Δ* double deletion strains with activation mutants for Fus3 (*FAM*), Kss1 (*KAM*), and Hog1 (*HAM*), revealing distinctive similarities of *FAM* and *fus3Δ*/*tor1Δ* yeast, specifically, and distinctive differences for similar comparisons with the other MAPK mutants. All results were determined from the same qCLS plate experiment. (**D**) Comparison of the survival decay rates (measured between day5-day17) for single gene deletion, *mapkΔ*/*tor1Δ*, and activation mutant strains grown in SD medium with 2% glucose. Error bars throughout the figure represent the standard deviation across 3 analytical replicate experiments.

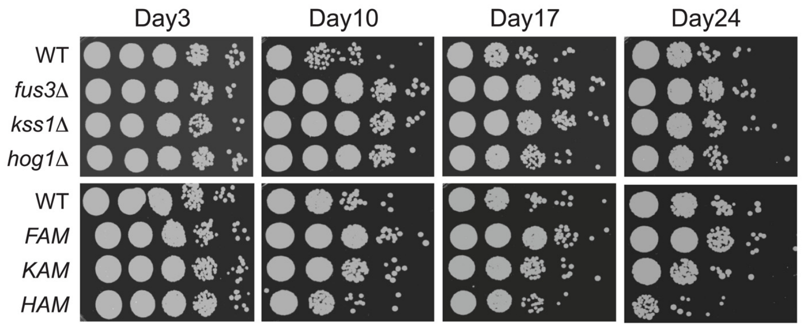

Figure S5. Qualitative CLS spot assay comparison of *MAPK* gene deletion versus activation site mutation. Qualitative CLS assays were conducted as described (*see methods and materials*) for *mapkΔ* (top) and activation site (bottom) mutants revealing generally consistent responses between qualitative and quantitative CLS assays.

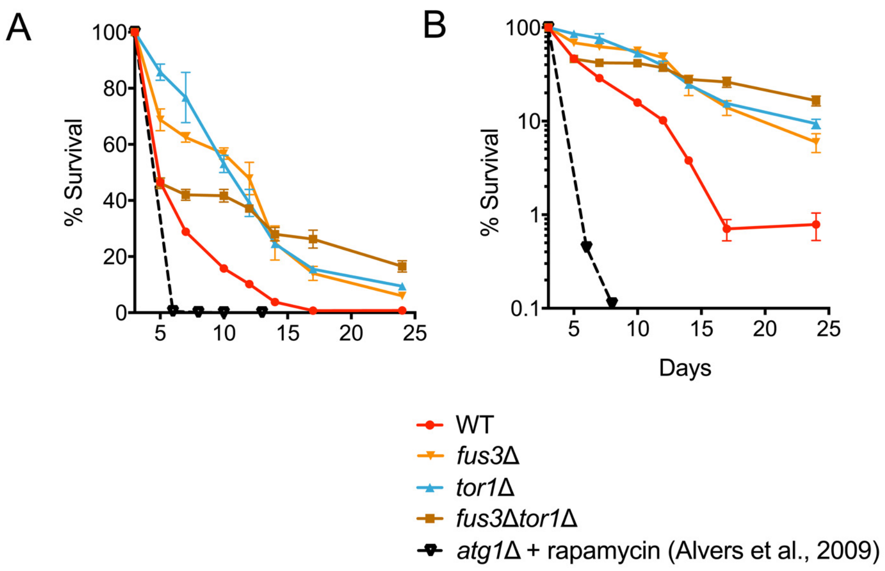

Figure S6. Experimental and modeled data for the comparative effects of autophagy-null, *tor1Δ*, and *fus3Δ/tor1Δ* cells. (**A**) Linear or (**B**) Log-scaled data from Figure 6A were overlaid with data extracted from Alvers et. al., in which they measured the CLS of *BY4742* yeast lacking autophagosomal mutants (*atg1Δ* shown here) in the presence or absence of rapamycin [16]. Absence of *ATG1* prevents autophagy from occurring at the early stages of chronological aging despite the inhibition of Tor1 by rapamycin, resulting in rapid survival decay that represents the fastest rate of decay possible for CLS [16]. In comparison, wild type and *fus3Δ/tor1Δ* cells exhibit a mid-point between autophagy-null (*atg1Δ*) and autophagy un-inhibited (*tor1Δ*) states.

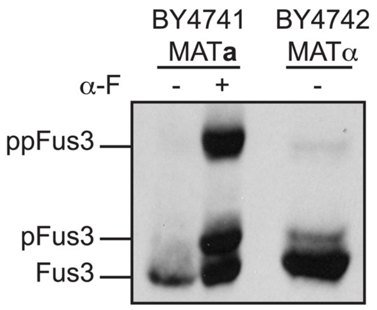

Figure S7. Fus3 is auto-activated in *BY4742* cells. Total cell extracts from log-phase BY4741 (MAT**a**) cells treated with and without α-factor mating pheromone (3 μM, 30 minutes) were compared to extract from log-phase *BY4742* cells using Phos-Tag SDS-PAGE and immunoblot analysis with anti-Fus3. Phos-tag gels retards the electrophoretic mobility of phosphorylated protein so as multiple phosphorylated states can be distinguished. The non-phosphorylated (non-activated; Fus3), mono-phosphorylated (partially activated; $Fus3^{pY182}$), as well as dual-phosphorylated (fully activated; $Fus3^{pT180,pY183}$) are made clearly visible upon treatment with pheromone in BY4741 cells. These same forms are also apparent in the absence of a pheromone stimulus in *BY4742* cells, suggesting that the phospho-activation sites of Fus3 are required for kinase activation and in turn promote Fus3 regulation of longevity control processes.
